# Interpretation and Visualization of Non-Linear Data Fusion in Kernel Space: Study on Metabolomic Characterization of Progression of Multiple Sclerosis

**DOI:** 10.1371/journal.pone.0038163

**Published:** 2012-06-08

**Authors:** Agnieszka Smolinska, Lionel Blanchet, Leon Coulier, Kirsten A. M. Ampt, Theo Luider, Rogier Q. Hintzen, Sybren S. Wijmenga, Lutgarde M. C. Buydens

**Affiliations:** 1 Institute for Molecules and Materials, Radboud University Nijmegen, Nijmegen, The Netherlands; 2 TNO Quality of Life, Zeist, The Netherlands; 3 Department of Neurology, Erasmus University Medical Centre, Rotterdam, The Netherlands; Governmental Technical Research Centre of Finland, Finland

## Abstract

**Background:**

In the last decade data fusion has become widespread in the field of metabolomics. Linear data fusion is performed most commonly. However, many data display non-linear parameter dependences. The linear methods are bound to fail in such situations. We used proton Nuclear Magnetic Resonance and Gas Chromatography-Mass Spectrometry, two well established techniques, to generate metabolic profiles of Cerebrospinal fluid of Multiple Sclerosis (MScl) individuals. These datasets represent non-linearly separable groups. Thus, to extract relevant information and to combine them a special framework for data fusion is required.

**Methodology:**

The main aim is to demonstrate a novel approach for data fusion for classification; the approach is applied to metabolomics datasets coming from patients suffering from MScl at a different stage of the disease. The approach involves data fusion in kernel space and consists of four main steps. The first one is to extract the significant information per data source using Support Vector Machine Recursive Feature Elimination. This method allows one to select a set of relevant variables. In the next step the optimized kernel matrices are merged by linear combination. In step 3 the merged datasets are analyzed with a classification technique, namely Kernel Partial Least Square Discriminant Analysis. In the final step, the variables in kernel space are visualized and their significance established.

**Conclusions:**

We find that fusion in kernel space allows for efficient and reliable discrimination of classes (MScl and early stage). This data fusion approach achieves better class prediction accuracy than analysis of individual datasets and the commonly used mid-level fusion. The prediction accuracy on an independent test set (8 samples) reaches 100%. Additionally, the classification model obtained on fused kernels is simpler in terms of complexity, i.e. just one latent variable was sufficient. Finally, visualization of variables importance in kernel space was achieved.

## Introduction

Currently, due to the increasing amount of data generated from different analytical platforms for a single studied system, for instance in fingerprinting a disease in the metabolomics and proteomics fields, optimal data concatenation, or data fusion, has become an issue that needs to be addressed. Each analytical technology demonstrates different strengths and limitations regarding its capability to distinguish between different biological conditions, depending upon factors such as sensitivity, sample preparation, analytical stability, and analytical reproducibility. The jointed use of two or more analytical technologies gives then a more robust strategy for data analysis than the use of a single platform [1].

Data fusion is widely applied in the pattern recognition field [2]. For example, in chemistry, biology, medicine and many others fields linear techniques are used to construct a mathematical model that relates spectral responses from different techniques to analyte concentrations [3], [4], [5], [6]. In the omics related fields, data fusion is performed in different ways and on different data levels [7]. To date, data fusion methods are organized in three levels: low-level, mid-level and high-level fusion [8], [9]. In low-level fusion, different data sources are concatenated at the data level. In the mid-level fusion, data from different sources are combined at the data level by selection of variables or at the latent variables level. In high-level data fusion, different model responses (for instance prediction for each available data set) are joined to produce a final response. Currently, several linear techniques, such as Principal Component Analysis (PCA) or Partial Least Squares Discriminant Analysis (PLS-DA), are used for the above mentioned types of data fusion. These different linear data fusion approaches have been applied with good success in recent times in the different omics fields, including metabolomics [8], [10], [11], [12]. To our knowledge non-linear methods have not been applied to data fusion in for instance metabolomics. However, some chemical systems and problems are inevitably non-linear and reveal characteristics in a non-linear fashion. The assumption of a linear response is then incorrect and non-linear description is appropriate [13]. Of course, to follow Occam’s razor principle, it is common practice to first apply linear methods and only if they fail to move to non-linear techniques like kernel-based methods. Kernel-based methods transform the data to a high dimensional feature space by means of a kernel function. This generates a new data matrix, which can be viewed as a similarity matrix. The kernel function takes relationships that are implicit in the data and makes them explicit, so that patterns are easier to detect. Moreover, they have been designed to deal with datasets where many variables are present. Kernel-based methods have already been demonstrated to form powerful tools and therefore are widely applied to various statistical problems due to their flexibility and good performance [14], [15]. A major disadvantage of these Kernel-based methods has been that information on the importance of variables is lost. However, recently an approach has been proposed for representing the importance of variables in kernel space, a method based on the principles of so-called pseudo samples [16], [17].

Nowadays, proton Nuclear Magnetic Resonance (^1^H-NMR) and Gas Chromatography-Mass Spectrometry (GC-MS) are well-established powerful analytical methods for generating metabolomics profiles. For analysis of complex, biological samples like those from Cerebrospinal fluid (CSF) both techniques have their advantages and disadvantages. For instance, ^1^H-NMR requires limited sample preparation, is quantitative, non-destructive and unbiased. ^1^H-NMR may detect compounds that are too volatile for GC, while metabolites without proton (phosphoric acid) are not detected by ^1^H-NMR. GC-MS requires derivatization and thus more time consuming sample preparation. On the other hand, GC-MS yields a higher sensitivity than NMR and therefore may detect metabolites that are present in a concentration below the detection limit of ^1^H-NMR. Therefore, these analytical platforms give wide and complementary views of the studied system. To obtain the maximum/optimal amount of relevant information about the complex biological system, the data from these powerful analytical techniques need to be combined and analyzed with advanced multivariate statistical tools.

This paper presents a novel framework for integrating data from different analytical sources by applying non-linear kernel-based statistical learning methods. We demonstrate this non-linear kernel fusion approach on ^1^H-NMR and GC-MS metabolomics datasets obtained from CSF of patients with Multiple Sclerosis (MScl) [18]. These data display non-linear response characteristics. The proposed approach for non-linear Kernel-based data fusion consists of four steps. The first step aims to extract relevant variables from both datasets separately. Variable selection is performed by means of Support Vector Machine Recursive Feature Elimination (SVM-RFE) for non-linear kernels [19]. The second step is designed to fuse the relevant information of both datasets by using linear combinations of kernel matrices [20]. This kernel fusion falls outside the range of the classical low-, mid- and high-level fusion. The next step (step 3) consists of applying PLS-DA on the fused kernels as classification method. In step 4, the visualization of the relative contribution of each variable to K-PLS-DA model (variable importance) was achieved by applying and extending the recently developed pseudo samples principle [16], [17]. Consequently, in our approach the importance of variables is visualized. The variables can then be interpreted in terms of the underlying biology of system. Application of our non-linear Kernel-based data-fusion methodology to the ^1^H-NMR and GC-MS metabolomic datasets from samples of CSF of MScl individuals and individuals in the early stage of the disease enabled better classification than using the data from the two sources separately. More importantly, the biological interpretation can now be done based on the joined data from the two platforms. The approach proposed here can be extended to other types of datasets such as to MS or NMR data from proteomics or data from microarrays and Liquid Chromatography. The number of samples used to study the progression of MScl is relatively small. Therefore, some limitations with respect to biological interpretation as well as prediction of future samples may exist, e.g. due to biological variation. In order to use the findings in the clinic they should be validated in a new cohort with a larger number of samples. This issue will be further addressed in the discussion section.

## Materials and Methods

### CSF Sampling and Patients

The CSF patients involved in this study were all followed by the Rotterdam Multiple Sclerosis Center and the department of Neurology at Erasmus University Medical Center (Rotterdam, The Netherlands). The Medical Ethical Committee of Erasmus University Medical Centre in Rotterdam, The Netherlands, approved the study protocol and all study patients gave written consent. All CSF samples were specifically collected from patients that were not under any drug treatment.

All CSF samples were taken from patients via lumbar puncture. Immediately after sampling, the CSF samples were centrifuged to remove cells and cellular elements (10 minutes at 3000 rpm). Subsequently, a fraction of the CSF samples were used for diagnosis purpose and the remaining amounts were aliquoted and stored at −80°C.

The CSF samples were classified into two groups. The first group consisted of CSF samples collected from patients diagnosed with MScl. The second group of CSF samples was taken from patients who were diagnosed with clinically isolated syndrome of demyelination (CIS), which represents an early stage of MScl. It is worthwhile to mention that all patients diagnosed with CIS have later developed MScl. The overview of the available CSF samples for NMR and GC-MS is presented in Table 1, while clinical information is described in File S1. It is important to mention that the set of samples analysed by NMR and GC-MS only partly overlap (Table 1).

**Table 1:** The number of samples included in a training and independent test set.

Group	No. samples NMR			No. samples GC-MS			Overlap NMR and GC-MS		
	Training	Test	Total	Training	Test	Total	Training	Test	Total
MScl	19	7	26	18	6	24	7	5	12
CIS	15	5	20	10	4	14	7	3	10

### NMR Samples Preparation and Data Acquisition

The CSF samples of the CIS and MScl classes were prepared as follows. An aliquot of 20 µL of the stored frozen human CSF sample (−80°C) was thawed at room temperature. Subsequently, 200 µL D_2_O was added to biofluid in order to obtain sufficient sample volume for NMR measurements. We used 3-(Trimethylsilyl)propionic-2,2,3,3-d_4_ acid sodium salt (TSP-d_4_ 99 at.%D) as internal standard for chemical shift reference (δ 0.00 ppm) and metabolite quantification. For this and buffering, 70 µL of buffer solution was added to the 220 µL of human CSF sample. The buffer solution solvated in a mixture of water and D_2_O consists of 2,85 mM TSP, 6.92 mM sodium azide (NaN_3_) and 42.08 mM sodium phosphate dibasic dehydrate (Na_2_HPO_4_•2H_2_O). The addition of mixture solution to 220 µL of CSF sample leads to a final concentration of 0.66 mM TSP-d_4_ and corresponding concentrations of buffer solution components. The pH of the CSF NMR sample was adjusted to around 7 (7.0–7.1) by the buffering capacity of the phosphate in the buffer solution. The final CSF NMR sample (290 µL) was transferred to a SHIGEMI microcell tube for NMR measurements.

All spectra were recorded by using a standard pulse sequence (1D-NOESY; recycle delay-90°-t1-90°-t_m_-90°) at a temperature of 25°C. The water suppression was achieved by presaturation during the relaxation delay (8 s) and mixing time (100 ms). All ^1^H NMR spectra were acquired at 600 MHz Bruker NMR Spectrometer equipped with cryo-cooled probe. For each 1D ^1^H NMR spectrum 256 scans were accumulated with a spectral width of 7200 Hz resulting in a total of 16K data points. The acquisition time for each scan was 2.2s. Prior to spectral analysis, all Free Induction Decays (FIDs) were multiplied with a 0.3 Hz line broadening function, Fourier transformed and manually phased. In addition, the TSP internal reference peak was set to 0 ppm. This initial processing was done using ACD/SpecManager software version 12.02 [21].

All 46 human CSF spectra were acquired and pre-treated as described above and subsequently, transferred to Matlab, version 7.6 (R2008b) (Mathworks, Natick, MA) for further analysis.

### Preprocessing of NMR Spectra

The NMR spectral data of human CSF was pre-processed, which typically involves baseline correction, alignment, binning, normalization and scaling. Asymmetric Least Square method was used for baseline correction of NMR spectra [22]. Next, in order to remove variations in peak position, NMR spectra were aligned by using correlation optimized warping [23]. A further problem is the high dimensionality of the data (*circa* 15000 variables). To reduce the number of variables associated with the NMR spectra, we performed binning via adaptive intelligent binning [24]. Before binning data were normalized to total area. The chemical shift ranges of δ 0.75–4.15 and δ 8.65–8.85 were used for the binning procedure. The binning procedure led to 233 bins in total. In the final step of preprocessing data were scaled to unit variance.

### GC-MS Samples Preparation and Data Acquisition

The GC-MS method applied here is a non-targeted GC-MS method which uses a derivatization step that has frequently been applied for metabolomics studies [25]. With this method it is possible to analyse simultaneously various classes of (polar) metabolites, e.g. amino acids, organic acids, fatty acids, sugars.

Human CSF samples (100 µL) were deproteinized by adding 400 µL methanol and subsequently centrifuged for 10 min at 10000 rpm. The supernatant was dried under N_2_ followed by derivatization with methyl-N-(trimethylsilyl)-trifluoroacetamide (MSTFA) in pyridine similar to Koek et al. [25]. During the different steps in the sample work-up, i.e. prior to deproteinization, derivatization and injection, different (deuterated) internal standards were added at a level of circa 20 ng/µL. The end volume was 135 µl and 1 µl aliquots of the derivatized samples were injected in splitless mode on a HP5-MS 30 m×0.25 mm×0.25 mm capillary column (Agilent Technologies, Palo Alto, CA) using a temperature gradient from 70°C to 320°C at a rate of 5°C/min. GC-MS analysis was performed using an Agilent 6890 gas chromatograph coupled to an Agilent 5973 quadrupole mass spectrometer. Detection was carried out using MS detection in electron impact mode and full scan monitoring mode (m/z 15−800). The electron impact for the generation of ions was 70 eV.

A total of 38 human CSF samples were analysed by GC-MS. The samples were randomly distributed over batches and each sample was injected once. A pooled CSF sample was prepared from the study samples for quality control (QC). Aliquots of this QC sample were analysed in sextuplicate in each batch according to the procedure described by van der Greef et al. [26].

Data preprocessing was performed by composing target lists of peaks detected in the samples based on retention time and mass spectra. Peaks were characterized by retention time and *m*/*z* ratio and identified by comparison with a spectral database. These peaks were integrated for all samples. The peak areas were subsequently normalized using internal standards and corrected for intra- and inter-batch effects using the QC samples according to the procedure described by Verheij et al. [27]. The final step of preprocessing was unit variance scaling.

### Explorative Analysis

The first step of our data analysis strategy consists of a data exploration by means of Robust – Principal Component Analysis (R-PCA) [28] and PCA. R-PCA was employed on the autoscaled data to detect the outliers in both datasets. To extract and display the systematic variation in the two datasets PCA was also carried out on the autoscaled data.

### Selection of Training Set and Independent Test Set

In order to validate the performance of the classifier an independent test set was used. Dividing the data into training and test sets is a widely accepted approach for this purpose [29], [30]. The commonly used leave-one-out cross-validation (LOOCV) is biased to assess the predictive ability of the classification model. External validation using test sets provides a means to establish a more reliable predictive performance of the classification model [31], [32], [33].

The training set and an independent test set were selected separately for NMR and GC-MS datasets using the Kennard-and-Stone algorithm [34] in such a way that the number of samples in the test set in every group (i.e. MScl or CIS, see Table 1) was equal to 25% of the total number of samples in a group). The training sets were used for all optimization steps and for developing a classifier, while the independent test was utilized to assess the predictive ability of the classification model. The Kennard-and-Stone algorithm is one of many possible approaches for data division [32], [35], [36]. The use of Kennard-and-Stone algorithm for data division is justified by the advantage of obtaining representative training set and the reproducibility of the selection. Nevertheless, since it is Euclidean based algorithm it might be influenced by noisy variables. Therefore, in addition the training and independent test sets were selected randomly. The results of presented fusion approach for random division is shown in Figure S3 and File S1.

The number of samples included in the training set and independent test set is shown in Table 1. Since the number of overlapping samples between NMR and GC-MS is relatively low (22) this puts limitations on the accuracy of the predictions when using a relatively small independent test. Therefore, as an additional check of the meaningfulness of the classification model, a permutation test with 10000 permutations was performed. Using a permutation test, we checked if the assessment of the classification of objects into the original classes is significantly better than any random classification in two arbitrary classes.

### Supervised Analysis: Linear and Non-linear Approaches

The supervised analysis is carried out in order to extract class related information. Below, we briefly describe the overall strategy. First, the supervised data analysis involving linear methods is described followed by the proposed kernel based non-linear methods. In the next sections detailed information on specific technical aspects of the supervised data analysis strategy is provided.

The most straightforward approach in data analysis is to first use a linear method. Therefore, the linear method by means of the cross model validation (CMV) PLS-DA is applied [37]. In this technique, two cross validation procedures are included in the variable selection procedure based on jack-knifing. This approach enables removal of irrelevant variables and optimizes the model for accurate prediction of group memberships. This technique was first applied to individual datasets and then the selected variables were fused and analysed by the linear classifier.

**Figure 1:**
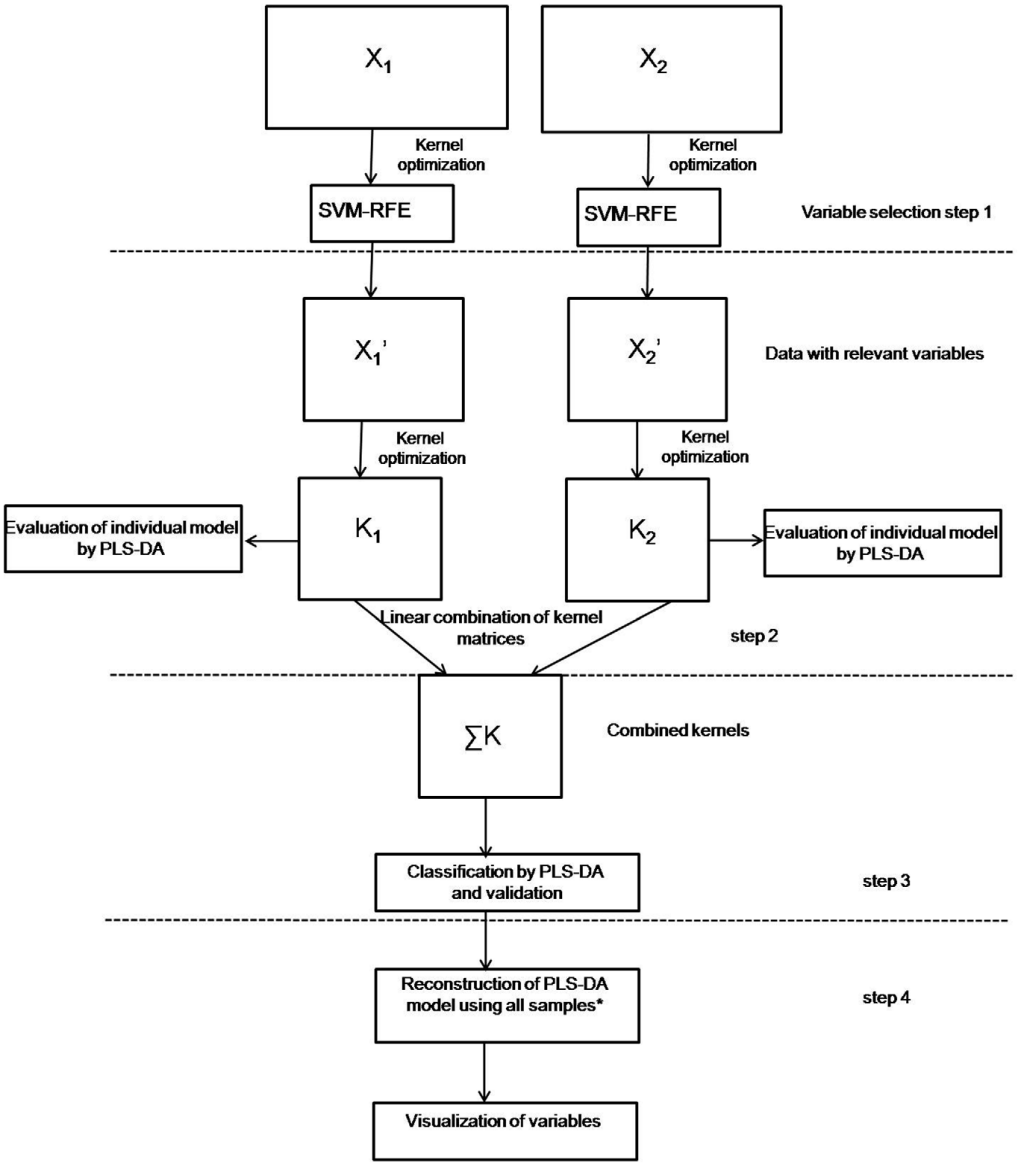
Conceptual flowchart of kernel-based data fusion. X_1_ and X_2_ are two blocks of data. *Note that all optimized parameters, i.e. number of variables, sigma for the rbf kernel, coefficients µ and nr. of LV’s are kept during the model reconstruction using all available samples. The particular steps are described in sections data analysis.

Next, if the considered classification problem is suspected to be non-linear (e.g. when prediction accuracy of linear model is low), more sophisticated algorithms can be applied. Here, a non-linear technique based on kernel methods was utilised. The strategy is shown in Figure 1. The steps 1 and 2 were carried out on the training set. The first step consists of a variable selection method, which aims to obtain meaningful information from each individual data set. We used SVM-RFE for the non-linear kernel as variable selection method [19]. For both datasets the radial basis function (rbf), i.e. a Gaussian function, is used to map the original input data into a feature space [38] (see also File S1 Kernel transformations part). The choice of kernel function is performed both by means of visual inspection of PCA score plot and using the root mean square error of cross validation (RMSECV).

RMSECV  =  
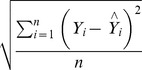
Here, Y is a real class label, while 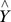
is the predicted class label; n indicates the number of observations.

The kernel parameter sigma (σ) is optimized by LOOCV. More specifically, in each iteration, one object from the training set is removed and a model is constructed on the remaining objects for different values of σ. This is repeated until each object has been removed once. The RMSECV is calculated for each iteration. The optimal σ value is selected based on the first minimum in the RMSECV. SVM-RFE is performed for both datasets separately. Only the selected variables are then used in the subsequent steps. In the final part of step 1 (see Figure 1), the data with only significant variables are analysed by K-PLS-DA, which is an alternative to the SVM technique. This part is employed to tune the kernel parameters and to estimate the classification accuracy of the separate datasets. The optimal model complexity (i.e. number of latent variables (LV’s)) was selected based on RMSECV. Note that the selected variables per dataset can be concatenated in classical mid-level fusion and analysed with K-PLS-DA. [8] In our procedure, SVM-RFE was selected as variable selection and K-PLS-DA as classification method. The use of SVM-RFE it justified by the fact that it is a well-established method, able to find significant variables in non-linear space. The binary classifier (PLS-DA) is a popular alternative to SVM. Our choice was guided by the fact that SVM offers sparse solutions based on a limited number of observations, i.e. the support vectors. Since the obtained hyperplane can be based on outlying objects, this brings a question about the robustness of SVM [38]. The main benefits of K-PLS-DA are its efficiency and simplicity. In addition, it has convenient visualisation options in the latent variable space. Nevertheless, as it will be shown latter, in terms of prediction K-PLS-DA and SVM perform similarly (see Data fusion by MKL).In the second step (see Figure 1), the kernels of the individual datasets are concatenated by linear combination of their kernel matrixes. In step 3 the combined kernels are analyzed with K-PLS-DA. The accuracy of the K-PLS-DA model is validated by the independent test set and by the permutation test. In order to obtain more robust classification model in the final step (number 4) the K-PLS-DA model is reconstructed using all available samples (i.e. both training and test sets) and all previously optimized parameters, namely number of variables, sigma for rbf kernel, coefficients µ and nr. of LV’s (see later). Moreover in this step variable importance in kernel space is evaluated and visualized.

### Variable Selection by SVM- RFE

The first step of our approach (Figure 1) consists of extracting the most relevant information from the datasets by using SVM- RFE variable selection. SVM is a powerful, supervised method and since this technique is extensively discussed in the literature we do not focus on its description [39]. SVM- RFE is an application of RFE in the SVM algorithm and was introduced by Guyon [19]. RFE is a backward elimination algorithm that ranks variables on the basis of the smallest change in a cost function minimized in the SVM algorithm. In the specific case of a non-linear kernel, used in this manuscript, the cost function to be minimized takes the form:
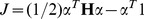
(1)Here, **H** is a matrix with elements y_i_y_j_ **K**(**x**_i_,**x**_j_), **K** is a kernel function, y_j_ and y_j_ denotes the class labels, α’s are the Lagrangian multipliers and 1 is a vector of ones. The algorithm begins by using all training data to train SVM. The matrix **H** is than recomputed for every variable being removed, while the α’s values remain unchanged. The elimination of the input variable “*i”* causes the change in cost function, J. The change in the cost function is calculated according to equation 2:
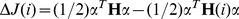
(2)Here, H(-i) indicates the matrix H calculated when the input component “i” is removed. All the ΔJ(i) is calculated and the values are sorted accordingly. A subset of variables corresponding to the end of the sorted list of ΔJ (i.e. those with small ΔJ) is then removed. In our case, the subset is formed by only one variable in each iteration.

In order to select an optimal set of variables LOOCV approach is used. We used RFE with cross-validation since it increases the likelihood that relevant variables are selected. Averaging over cross-validation iterations ensures that the variables that were significant in each run are selected. This gives a better estimation of the important variables than performing variable selection only once using all training samples. Moreover, using a variable selection procedure with cross-validation, overly optimistic results (solely valid for the training models) can be avoided. In LOOCV in each iteration, one object of the training set is left out and a ranking is obtained. Next, the total ranking is obtained by sorting the variables based on the amount of times it is selected in the LOOCV. All variables that appear twice or more in the “top ten” of the rankings are selected. Although the number ten is somewhat arbitrary, exploration of other options (e.g. “top fifteen” or median +1 of the amount it is selected in the LOOCV) did not affect the outcome.

### Data Fusion by Multiple Kernel Learning

The second step of our approach (step 2, Figure 1) aims to combine the kernels. This is done by means of Multiple Kernel Learning (MKL), which was pioneered by Lanckriet *et al.* [40]and Bach *et al.* [41] as extension of single kernel to integrate multiple kernels in SVM. They integrated multiple kernels in classification problems. The essence of MKL is to combine kernel matrices into a single kernel using basic algebraic operations such as addition or multiplication. For example, given two (positive semi-definitive) kernels **K_1_** and **K_2_** it is possible to define the new kernel **K**, which is a parameterized linear combination of **K**_1_ and **K_2_**. In particular, given a set of kernels **K** it is possible to combine them by linear combination according to equation 3:
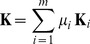
(3)Here m is a number of kernels and coefficients µ are non-negative to assure positive semi-definiteness of **K**: µ_i_ ≥0. Note that the dimensions of the kernels have to be equal (i.e. the number of samples in the datasets has to match). The coefficients µ_i_ in equation 3 can be tuned to weight the importance of the different kernels. The weights can be obtained in multiple ways, i.e. by applying different regularization method such as the L_1_ or L_2_-norm. L_1_ regularization on the kernel coefficients corresponds to the requirement that the sum of µ_i_ equals one (||µ_i_||_1_ = 1). L_1_ regularization can lead to sparse optimal solution and diminishing one of the platforms. In the current problem of deriving metabolite profiles from NMR and GC-MS datasets both datasets are relevant and complementary (the sets of measured metabolites are partially different). In order to avoid the possibility of shrinking the importance of any platform the L_2_-norm was used as regularization parameter. In the L_2_-norm approach, different constraint on the coefficients are used, i.e. the sum of squares of µ_i_ equals one (||µ_i_||_2_ = 1). The L_2_-norm yields a non-sparse solution and it distributes the coefficients over multiple kernels [20]. Using the L_2_-norm MKL we try to find the best separation between classes by solving the objective as follows:



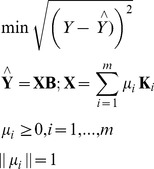
(4)The weights µ_i_ were optimized by LOOCV performed on the training set. The optimal weights were selected based on the minimal error of the root mean square error of cross-validation (RMSECV).

### K-PLS-DA and Variables Visualisation

In the non-linear architecture presented in Figure 1, the fused kernels are analyzed with a classification method, K-PLS-DA (step 3). This means that PLS-DA is applied on to the combined kernel matrix.

The classification model is statistically validated, i.e. it is based on the prediction accuracy of the K-PLS-DA model, on the independent test and on the permutation test. Therefore, in the final fourth step of the approach shown in Figure 1 K-PLS-DA is first reconstructed using all available samples and next variables importance is established.

**Figure 2:**
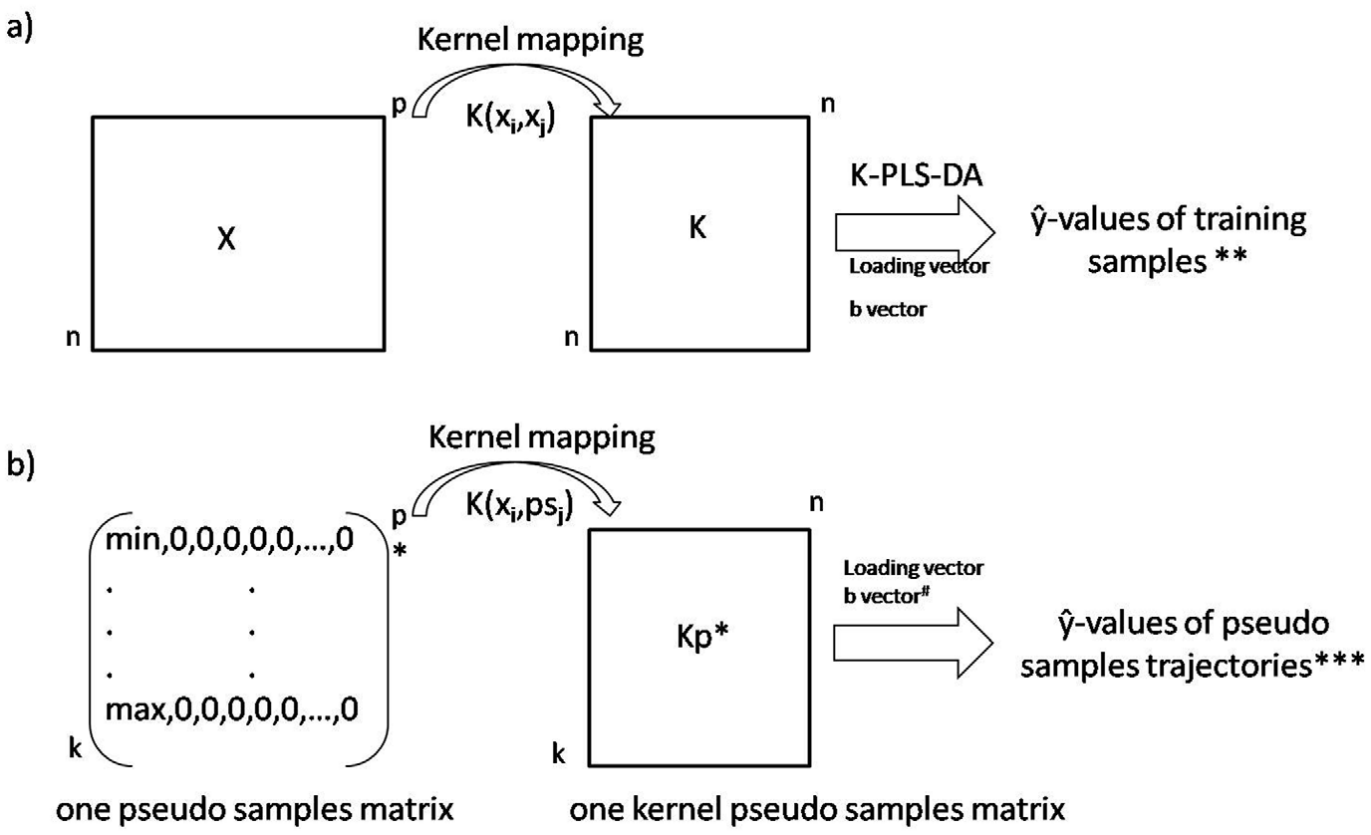
Representations of the a) kernel mapping of data matrix X into kernel space; b) pseudo samples principle in K-PLS-DA. k indicates the range of pseudo sample values (uniformly distributed); *Note that there are “p” pseudo sample matrixes and “p” kernel pseudo samples matrixes. **The ŷ-values can be projected into latent variable space. ^#^Note that for kernel pseudo samples the loading and **b** vector of K-PLS-DA model are used. ***These ŷ-values can be represented as “regression coefficients” shown later in Figure 4 or loading plot shown in Figure 5.

To represent the importance of the original variables, the pseudo samples principle, recently proposed by Krooshof *et al.* [16] and based on the non-linear plot principle described by Gower [42], was applied (step 4; Figure 1). As shown in Figure 2a, the matrix **X** (with n number of objects and p number of variables) is mapped by the kernel function K(x_i_,x_j_) (where x_i_ and x_j_ are samples from matrix **X**). The obtained kernel matrix **K** is a square matrix of size “n x n” (where n is a number of samples). The application of PLS-DA on the kernel matrix leads to a linear model, i.e. **y** = **Kb**+**r**, where **y** a vector of group memberships, **b** regression coefficients and **r** a model residual. It is possible to obtain predicted ŷ-values for all training samples of matrix **X,** but the information about the variables (i.e. metabolites) involved in the discrimination is lost. In our approach every original variable is represented as a set of pseudo samples. The pseudo samples are artificial samples constructed as follows: every pseudo sample contains a certain value (e.g. 1) for only one variable and zeros for all the others. It is possible to check the influence of these pseudo samples in a K-PLS-DA model by predicting their corresponding ŷ-values or projecting them into latent variable space.

The graphical representation of the pseudo samples principle is shown in Figure 2b. It is possible to construct for each original variable a series of pseudo samples containing different values. These different values permit to describe a complete trajectory for each variable. In that way, for every variable a matrix of size “k×p” (where k is the number of pseudo samples used to span the complete range of the original variable and p the number of original variables) is created. From now on, we call this set of pseudo samples describing a single original variable a pseudo samples matrix. For data matrix **X** (shown in Figure 2a) “p” pseudo samples matrices are created, each of size “k×p”. Once all pseudo samples matrices are constructed, one can apply the K-PLS-DA model to estimate the influence of the original variables. The pseudo samples are first mapped into the kernel space in relation to the original data matrix **X** using the same kernel function as derived for data matrix **X** (Figure 2a), i.e. K(x_i_,ps_j_) where x_i_ is an object of matrix **X** and ps_j_ is one pseudo sample. This leads to “p” kernel pseudo samples matrices (Figure 2b). Next the ŷ-values of pseudo samples can be estimated using regression vector “**b**” of K-PLS-DA model or they can be projected into LV space using loading vector of K-PLS-DA model. It has been shown that for linear kernel predicted ŷ-values of pseudo samples can be directly related to the regression coefficient of the original variables [17]. The projections of pseudo samples into the regression vector “**b**” of K-PLS-DA model from now on will be called “regression coefficient”; while the projection of the pseudo samples in the LV space will be referred to as a loading plot.

**Figure 3:**
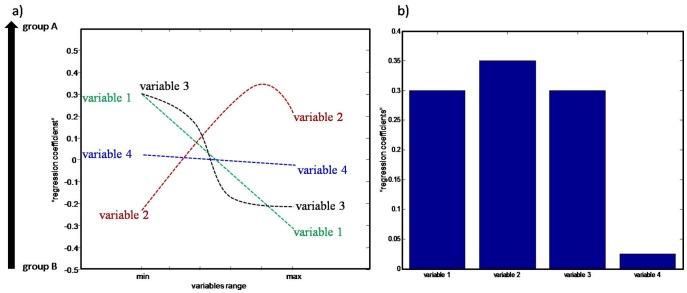
Schematic example of: (a) “regression coefficients” of original variables trajectories plotted versus their range; (b) the maximum absolute value of “regression coefficients” of original variables trajectories shown in a.

The first graphical representation (Figure 3a) permits one to investigate how the original variables evolve as a function of the studied response as well as their global and local importance in the model. As described above, the kernels pseudo samples (see Figure 2b) are projected into the K-PLS-DA model to visualize the importance and behaviour of the original variables. A schematic example is provided in Figure 3a. The “regression coefficients” of four variables trajectories are displayed, each one illustrating a different case. If the influence of a given variable to the model is linear the corresponding pseudo samples trajectory should form a straight line, as variable 1 in Figure 3a. Variable 2 behaves linearly in the low variable range but becomes non-linear in the high range as can be observed from the corresponding curvature. Variable 3 represents a more complex sigmoid shape. This variable has big importance in the model in the low range and in high range but less in intermediate range. Note that in the high range variable 3 shows a plateau, which indicates that after passing a certain concentration value its importance stays constant. Finally, the last variable shown in Figure 3a, variable 4, has very little influence on the model. Note that, if the optimal K-PLS-DA model complexity is one LV, information contained in the regression coefficient and the loading vector (obtained from K-PLS-DA model) is equivalent. Therefore it is possible to use the loading vector instead of the regression vector “**b**” for obtaining the predicted ŷ-values of pseudo samples (the y-axis of Figure 3a represent 1LV). This kind of plot in linear PLS-DA is called loading plot. Therefore, in the rest of paper it will also be called loading plot.

Another piece of information delivered from Figure 3a, is the change of variable value between studied groups. Positive predicted ŷ-values of pseudo samples indicates group A and a negative indicates group B. For instance the value of variable 1 increases from group A to group B, while the value of variable 2 decreases from group A to group B.

Figure 3b is an enhanced version of the figure presented in reference [17]. It allows direct visualisation of the importance of each variable on the K-PLS-DA model. Figure 3b is constructed as follows: the absolute value of the maximal “regression coefficients” (i.e. predicted values of pseudo samples) is used as the relative importance of each variable. Note that this approach can be used when the original variables are scaled to unit variance. Note further, an alternative to estimate/visualize the relative importance would be by taking the absolute value of the difference between maximum value and minimum value. The result can be graphically represented using the traditional regression plot obtained in any regression method [8]. Note that the importance of the variables can be also directly read off from Figure 3a, i.e. from the absolute max values along the horizontal axis. The 4 variables in Figure 3b correspond thus to the ones shown in Figure 3a.

### Data

Every NMR spectrum of CIS and MScl groups was divided into 233 bins, corresponding to relative metabolites concentrations. These bins are equivalent to approximately 50 identified metabolites and some unidentified resonances. The GC-MS data consists of 66 metabolites and their corresponding relative concentrations. It is important to mention that 20 metabolites were measured by both NMR and GC-MS. Some metabolites are identified only by NMR (e.g. methanol) or only by GC-MS (e.g. urea) [43].

These two datasets are used as case study to represent the proposed architecture for non-linear data analysis and fusion. After variables selection by SVM-RFE the NMR data and GC-MS data are reduced to 47 bins and 29 metabolites, respectively. In case of NMR these 47 informative bins correspond to 20 identified metabolites and some unidentified resonances.

It is important to keep in mind that σ, i.e. the parameter controlling the smoothness of the function, has to be tuned correctly, since it impacts the model performance. The σ parameter used for rbf kernel function is optimized separately for NMR dataset and GC-MS dataset and again before kernel fusion. An overview of σ parameters optimized in particular steps in Figure 1 is summarized in Table 2.

**Table 2:** Summary of σ parameter for rbf kernel function.

σ parameter at:	NMR	GC-MS
Step 1 (variable selection)	0.5	0.55
Step 3 (kernel fusion)	0.3	0.3

### Metabolites Identification

After selection and visualization of the most important variables, the corresponding metabolites were identified (NMR). Metabolite identification for NMR data was carried out by using the 600 MHz library of metabolite NMR spectra from the Chenomx NMR Suite 7. The library of metabolite spectra is obtained based on a database of pure compound spectra acquired using particular pulse sequence and acquisition parameters, the tn-noesy-presaturation pulse sequence with 4s acquisition time and 1s of recycle delay [44].

## Results

### Linear Methods

The analysis of the data can be first performed per analytical method. This is particularly significant not only during exploratory phase but also during supervised analysis, where relevance of individual sets is investigated. Both datasets were first analyzed with R-PCA and PCA for presence of outliers and to detect potential trends. In total 4 NMR spectra and 3 GC-MS samples were detected as outliers and removed from further analysis. Since PCA score plots did not reflect any groupings and the variations did not separate according to groups CIS and MScl, the results of this analysis are not shown. Next, the linear method, CMV-PLS-DA, was employed on separate platforms and on fused datasets in mid-level fashion. In our case, the application of linear methods provided disappointing results for the separate datasets as well as for the datasets fused in the mid-level fashion. The degree of correct classification for a validation set obtained for the individual data-set analysis and for the concatenated sets can be seen in Table 3 and the corresponding figures are shown in Figure S2a−S2c.

**Table 3:** An overview of prediction accuracy for the validation set using linear methods, non-linear methods and MKL.

	Correct classification rate		
	NMR	GC-MS	Fusion(NMR +GC-MS)
Linear method	61%	63%	65%
Non-linear method	93%	85%	89%
MKL			100%

### Non-linear Analysis

Since linear methods did not lead to satisfactory results (see Table 3), we used more sophisticated methods (i.e. non-linear) to find differences in metabolic profiles of CSF of CIS and MScl groups. As pointed out previously (see Materials and Methods), our approach is based on four steps. The first one consists of a variable selection performed on each dataset. We used SVM-RFE in order to get good predictive group membership ability and a meaningful interpretation of the model. After the first step, we analysed the separate datasets by K-PLS-DA. After variable selection every dataset can be assessed in terms of complexity and prediction accuracy. The overall correct classification for independent test sets, left out during model optimization and construction, is 93% for NMR data and 85% for GC-MS data, respectively (Table 3). Both K-PLS-DA models were constructed for 2 latent variables (LV’s). These results suggest that both data sources hold relevant information concerning discriminating CIS and MScl groups. The overview of the prediction of each K-PLS-DA model is presented in Table 3.

The most straightforward approach for data fusion is to analyse the two datasets together by simply concatenating the selected variables from two data sources together (mid-level fusion). It is expected that the two types of information from the NMR and GC-MS datasets should complement each other and improve the class separation. However, this mid-level fusion provides very similar results in terms of complexity of the K-PLS-DA model and correct classification (i.e. 2LV’s and 89% correct classification, see Table 3).

### Data Fusion by Multiple Kernel Learning

Since the analysis of both sets as unique matrices does not provide a better separation of the groups, we decided to apply kernel-based fusion by MKL (Materials and Methods). Note that this kernel fusion architecture, as applied by us here, falls outside the range of the classical low-, mid-, or high-level fusion. It uses the specific property of the kernel matrix in the data fusion, i.e. its dimensions and its nature comparable to a similarity matrix.

The MKL approach used here is composed of optimizing weights for each kernel matrix. The optimized weights were equal 0.75 for NMR and 0.661 for GC-MS. This indicates that both datasets are almost equally important. After weighted kernel-based fusion, the newly formed kernel matrix can by analysed by PLS-DA. The kernel fusion leads to correct prediction of 100% on the independent test set (versus 89% for mid-level fusion, see Table 3). The K-PLS-DA model was constructed by using 1 LV. As an additional check, we performed a permutation test. The p-value for 10000 permutations was equal to 0.0013. The accuracy of K-PLS-DA was further compared to SVM. The correct prediction was as well 100% on the independent test set.

Since the model shows good predictive ability on the independent test set, we consider it as statistically validated and as shown in Figure 1 (step 4) the K-PLS-DA model is then reconstructed using all available samples. The resulting model can be graphically assessed using a score plot (here not shown). Obviously, this kind of plot is the normal visual representation of kernel method.

### Variables Importance Visualization

**Figure 4:**
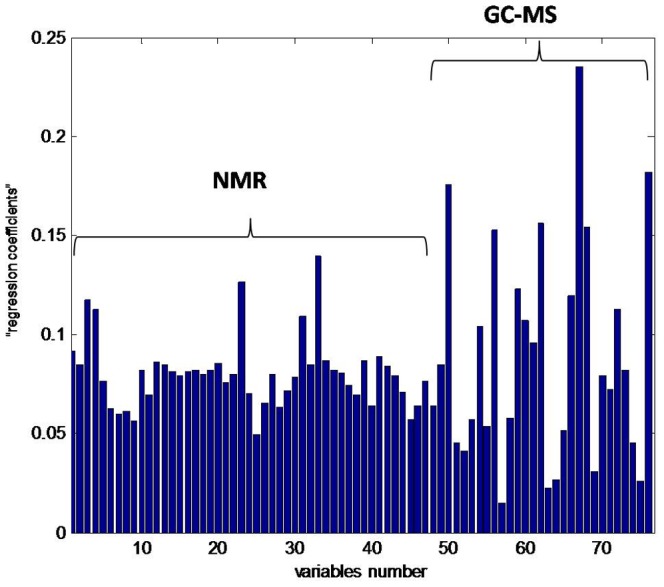
The maximum absolute value of “regression coefficients” of original variables.

As shown in Materials and Methods, it is possible to visualize the original variables in discriminating the groups. For that purpose the maximum absolute value of the predicted values of pseudo samples was calculated. The obtained values are shown in Figure 4. This figure demonstrates that there are several variables having very high importance. For instance, variables number 67 (sucrose), 76 (urea) and 50 (3-methyl-2-hydroxybutanoic acid) have the highest values of the predicted values of pseudo samples, demonstrating the relevance of these variables. There are just few variables seen as less significant, for example variable 57 (glycerol) or 63 (phenylalanine). The complete list of names of metabolites corresponding to variable number is given in Table S1.

**Figure 5:**
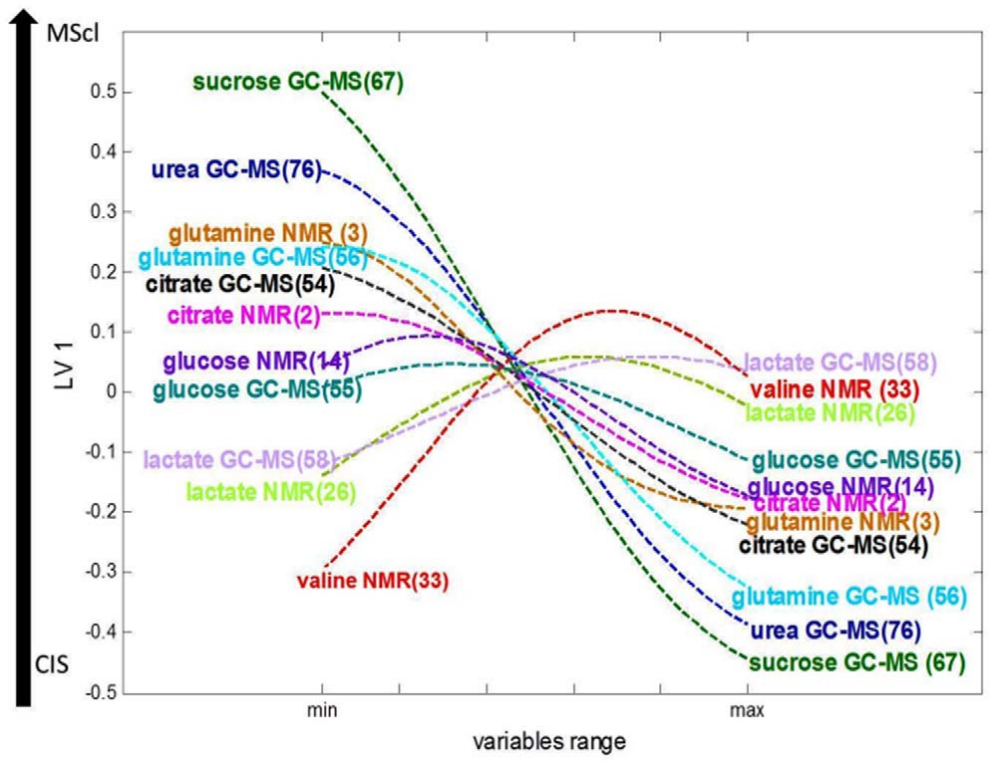
Loading plot of pseudo samples trajectories for selected variables. Numbers in the brackets correspond to variable numbers in Figure 4.

As was explained in Materials and Methods, to investigate the relation between individual variables and changes of metabolite concentration (i.e. elevation or reduction) the trajectory of predicted values of pseudo samples (representing individual variables) can be studied. Since the optimal model complexity is 1LV we used loading vector delivered from K-PLS-DA model to project the pseudo samples into LV1. The obtained trajectories are shown in Figure 5. Because presenting trajectories for all variables makes the plot unreadable, in Figure 5, only a few of them are given. Trajectories for all variables are given in Figure S1.

Besides showing the importance of variables in discriminating, Figure 5 also reveals the linear or non-linear trend and/or monotonicity of the variables in certain concentration ranges. A variable which shows a non-linear trend is glutamine and is derived from NMR. Valine is characterized by linearity and monotonicity in its low range, and non-linearity in its high range. Urea and sucrose demonstrate linearity over the whole concentration range.

As mentioned before the change in metabolite concentrations across groups can be assessed. The horizontal axis in the Figure 5 represents the range of every original variable (scaled to its min to max value). The levels of lactate and of valine both increase, while the concentration of glutamine and citrate is reduced with disease progression. To make the change of metabolite concentrations more evident we included the direction of groups along vertical y-axis. More specifically, the negative values of the predicted values of pseudo samples correspond to CIS and the positive values to MScl.

At this point one should remember that some metabolites were measured both by NMR and GC-MS. It is therefore interesting to check how the corresponding variables compare with each other. For instance, pseudo sample trajectories for glutamine derived from NMR and GC-MS reveal very similar evolution upon disease progression. Correspondingly, pseudo sample for lactate, glucose and citrate measured by NMR and GC-MS display comparable trajectories along concentration range. This suggests that even after non-linear transformation the same variables measured by two different analytical methods are correlated and demonstrate their akin behaviour.

## Discussion

In this paper we have described a procedure for kernel-based data fusion. We have demonstrated an application of the proposed procedure to the classification problem of metabolomics datasets of CIS and MScl individuals. We have proposed a framework based on four steps, where the first one is focused on optimization of individual datasets. This is relevant, since we want to make sure that accurate information extracted from both data sources is included in fusion. We applied the L_2_ MKL framework, demonstrated by Yu *et al.* for SVM, to K-PLS-DA, since it is characterized by the non-sparse integration of multiple kernels. Indeed, the optimization of the L_2_ norm showed that both datasets, i.e. NMR and GC-MS, are valuable for discriminating CIS and MScl individuals.

The application of SVM-RFE allowed one to reduce both datasets significantly and select a set of informative variables. The classification performance of K-PLS-DA performed on fused kernels was better than single analysis and common mid-level fusion. Additionally, the K-PLS-DA model was simpler in terms of complexity, i.e. just one LV was sufficient to obtain optimal classification model.

The visualization of the variables relative contribution to K-PLS-DA model was achieved by applying and extending the pseudo samples approach demonstrated by [16], [17]. This allowed us to show that even after non-linear kernel transformation two different analytical methods are consistent with the results. The pseudo samples trajectories of the same variable measured by NMR or GC-MS demonstrate very similar trends.

Several potential challenges remain in the proposed framework. One possibility is to apply it to larger datasets with more different sources, for instance lipids and metabolites.

Since the number of available samples was limited, the potential impact of over-fitting of statistical model must be considered. We used here an independent test set and permutation test, which yielded results that clearly show that over-fitting, is highly unlikely. Note that in individual analysis the total number of samples was larger than in the fused set.

Classification with different types of non-linear functions in the original space can be achieved using diverse types of kernels. Of course, the choice of kernel function has to be done beforehand. A correct selection of kernel function has a significant influence on classification accuracy. However, no rules can be defined. In our experiment different kernel functions were tried. The Gaussian kernel was chosen as the one to fit the data properly. If the sigma value is too small over-fitting can easily occur. It has also influence on pseudo samples trajectories. Small sigma value (e.g. 0.1) might lead to very non-linear and hardly interpretable trajectories.

The case study described here represents indeed a non-linearly separable problem. As was shown, linear methods gave poor classification performance. The class separation was possible after application of a non-linear kernel function and PLS-DA. Non-linearity is also visible in the pseudo samples trajectories. There are several variables that are characterized by curved trajectories. The curvatures of the trajectories illustrate the effect of the original data. Importantly, even if these trajectories are non-linear, they are still simple enough to be interpretable.

The example given on the metabolites analysis of CSF gave very interesting results. However the number of used samples was relatively low. It should then be pointed out that due to this small number of samples, this study may have several limitations. Obviously, the size of the training and testing sets has an influence on the accuracy assessment of the classification method. Small sample size may result in detecting only the largest differences. Indeed the data size and classification rate are correlated.

The bigger the groups size the more representative and robust the results become. It has been shown [45] that as the sample size is increased prediction accuracy overcomes local minima and next stabilizes and therefore become more reliable and accurate. Obviously classification performance of a classifier is influenced by the natural difficulty of the studied problem, however there are possibilities where the performance of a classifier is degraded because of small training cases. Therefore one should be aware that results (with 100% correct prediction on test set) shown here do not imply that all new samples will be always correctly classified (due to e.g. biological variance). Hence, more studies involving a larger cohort will be necessary to fully establish and assess the findings. However, despite the drawbacks of the study, it seems that validation with the independent test set and the permutation test set attest that the results are meaningful. In general the accuracy of the classification of objects into the original classes is significantly better assessed than any random classification in two arbitrary classes (p-value of 0.0013). Additionally the random division of data to training and test set was performed, showing that the average correct prediction over 10000 different runs supports our results.

From a biological point of view the metabolites having a relatively high contribution in the K-PLS-DA model, e.g. urea, glutamine, lactate, citrate, valine, are consistent with biological knowledge. These metabolites, described in this study, were previously found in relationship to the MScl [6]. They, therefore, provide a biological validation for the fusion of data. However, the full interpretation of the presented models in terms of biology still remains to be made. Therefore, future work will focus on the interpretation of newly detected metabolites and on highlighting pathways involved in the MScl disease process. The pattern defined by these variables must also be studied by itself and put into context in a system biology approach.

The kernel fusion approach presented in this paper assumes the dimensionalities of the kernels to be equal, i.e. the samples present in each dataset have to come from the same subjects at the same time points. Although, extending our method for missing values appears valuable and would be an interesting subject for further research.

## Supporting Information

Figure S1 **Loading plot of pseudo samples trajectories for: (a) variables 1 till 19; (b) variables 20 till 36; (c) variables 37 till 57 and (d) variables 58 till 76.** Numbers correspond to variable numbers in Table 1a.
(TIF)

Figure S2 **The PLS-DA score plot of: (a) NMR data; (b) GC-MS data; (c) fused NMR and GC-MS in mid-level fashion.**
(TIF)

Figure S3 **Average Receiver Operating Characteristics derived from K-PLS-DA for random division of data.**
(TIF)

File S1 **Clinical information, kernel transformations and the results of shown fusion approach for random division of data.**
(DOC)

Table S1 **Metabolites.**
(DOC)
