# Supplementary figures and images for: Interpretation and Visualization of Non-Linear Data Fusion in Kernel Space: Study on Metabolomic Characterization of Progression of Multiple Sclerosis
Source: PLoS One. 2012 Jun 8;7(6):e38163. doi: 10.1371/journal.pone.0038163 (PMC3371049; doi:10.1371/journal.pone.0038163)

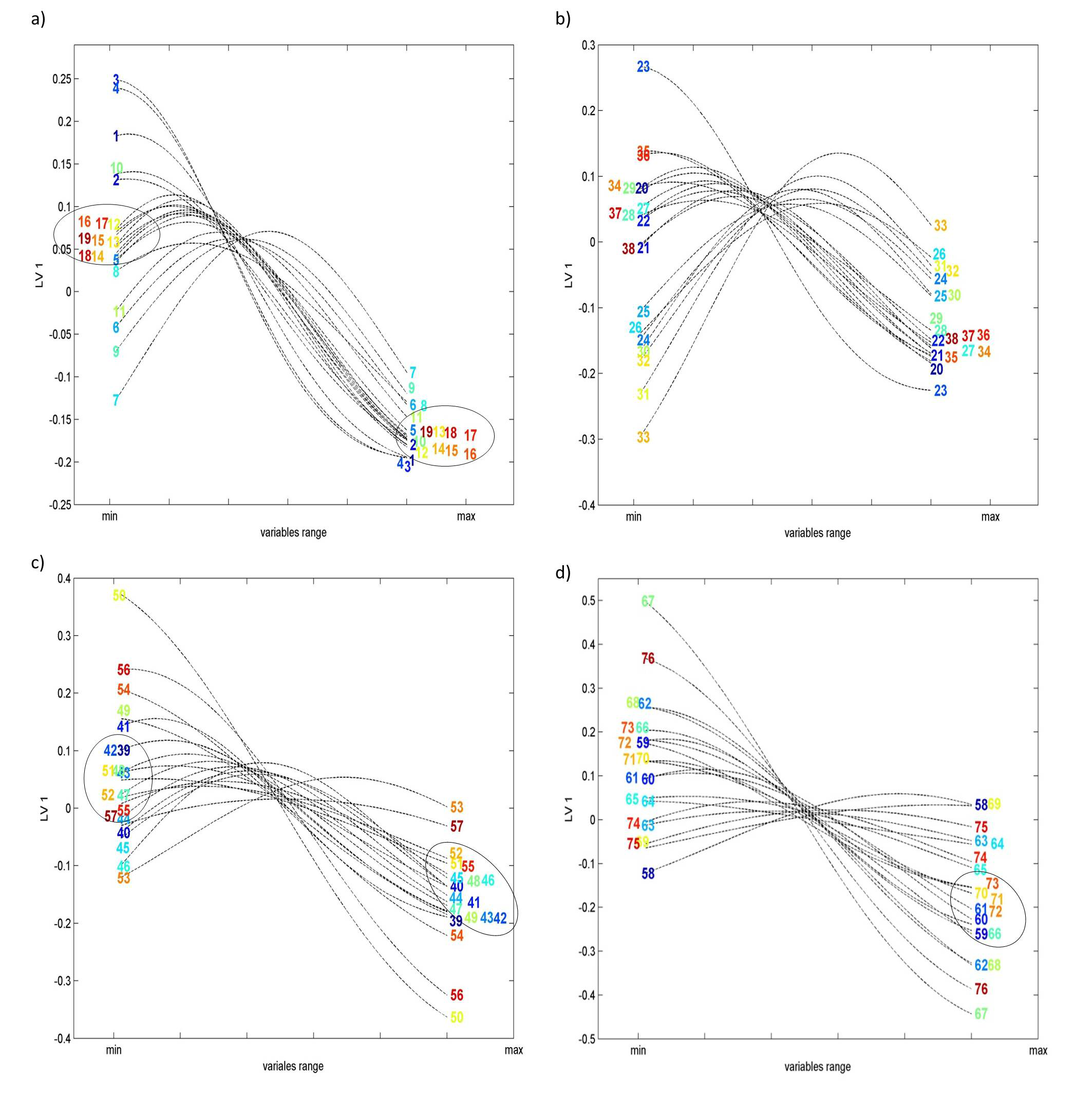

Supplement: Figure S1 — Loading plot of pseudo samples trajectories for: (a) variables 1 till 19; (b) variables 20 till 36; (c) variables 37 till 57 and (d) variables 58 till 76. Numbers correspond to variable numbers in Table 1a. (TIF) [file pone.0038163.s001.tif]

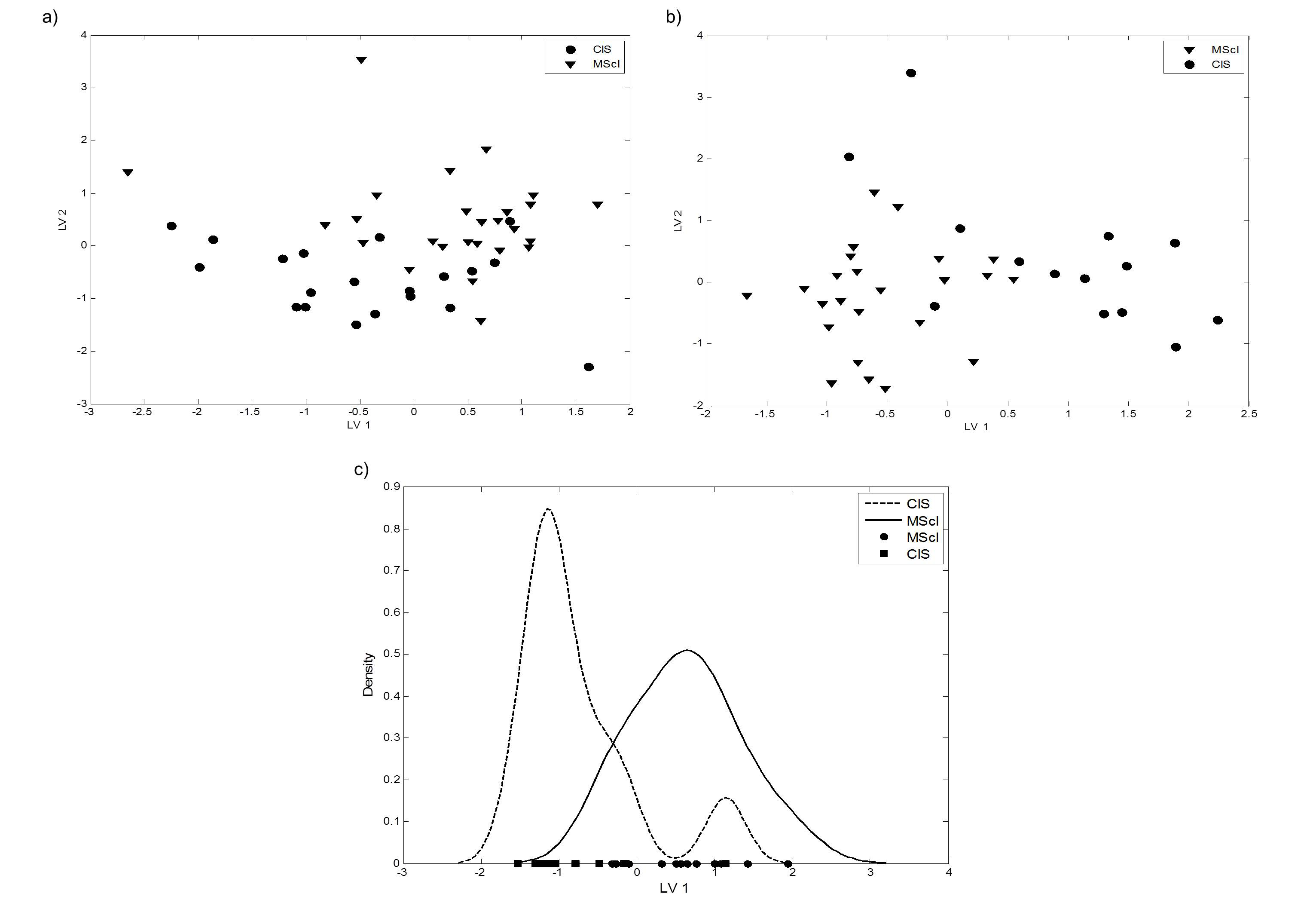

Supplement: Figure S2 — The PLS-DA score plot of: (a) NMR data; (b) GC-MS data; (c) fused NMR and GC-MS in mid-level fashion. (TIF) [file pone.0038163.s002.tif]

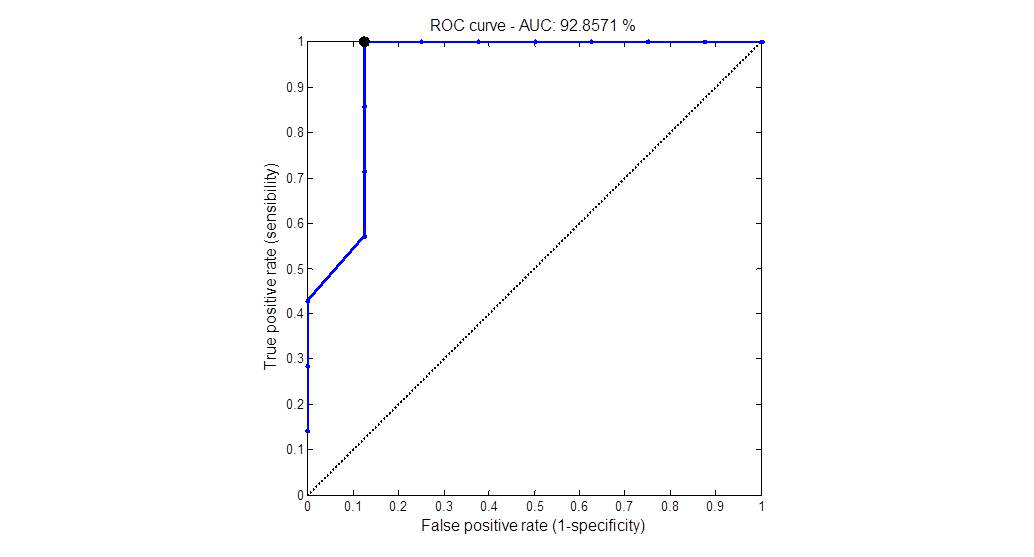

Supplement: Figure S3 — Average Receiver Operating Characteristics derived from K-PLS-DA for random division of data. (TIF) [file pone.0038163.s003.tif]
