# Supplementary material for: Interpretation and Visualization of Non-Linear Data Fusion in Kernel Space: Study on Metabolomic Characterization of Progression of Multiple Sclerosis
Source: PLoS One. 2012 Jun 8;7(6):e38163. doi: 10.1371/journal.pone.0038163 (PMC3371049; doi:10.1371/journal.pone.0038163)
Supplement: File S1 — Clinical information, kernel transformations and the results of shown fusion approach for random division of data. (DOC) [file pone.0038163.s004.doc]

**Clinical information**

Of the 26 patients in the MScl group in the NMR dataset, 19 patients had relapsing remitting (RR) MScl and 7 primary progressive (PP) MScl. In the GC-MS dataset of MScl patients, 7 patients were diagnosed with PP MScl and the others had PP MScl. The number of patients with PP MScl in the overlap NMR/GC-MS set is equal to 4. In the NMR dataset, the group of the MScl patients contains 6 males and 20 females, while in the GC-MS MScl dataset 5 males and 19 females are found. In the NMR/GC-MS overlap set (MScl) 4 patients are male. In the MScl NMR/GC-MS overlap set the median of the time of disease duration for the MScl patients at the moment of CSF sampling was 4.5 years, while the average was 7.25 years with standard deviation of 6.6 years. For the complete NMR MScl set and GC-MS MScl set the disease duration was on average 6 years with standard deviation of 5 years and 7.3 years with standard deviation of 5.7 years, respectively. In the group of 20 patients with CIS, 5 are males and 15 are females in the NMR set. In the GC-MS dataset 4 patients are male. It is worthwhile to mention that all patients diagnosed with CIS have later developed MScl.

**Kernel transformations**

Different kernels transformations, namely linear and polynomial (2^nd^ and 3^rd^ degree), were studied. However the correct classification did not extent 60% for independent test set. Moreover, these kernels transformation presented the lowest RMSECV for training set.

**Random division in training and independent test set**

Random division (repeated 10^4^ times) of the data was made and the MKL procedure was performed for each pair of training and independent test set. The average correct prediction of test set was equal to 90.5%. The average Receiver Operating Characteristics (ROC) curve of K-PLS-DA model is shown in Figure S3 with area under cure of 92.8%.
