# Supplementary material for: Interpretation and Visualization of Non-Linear Data Fusion in Kernel Space: Study on Metabolomic Characterization of Progression of Multiple Sclerosis
Source: PLoS One. 2012 Jun 8;7(6):e38163. doi: 10.1371/journal.pone.0038163 (PMC3371049; doi:10.1371/journal.pone.0038163)
Supplement: Table S1 — Metabolites. (DOC) [file pone.0038163.s005.doc]

Table S1.

| Variable nr. | Metabolite | Variable nr. | Metabolite |
| --- | --- | --- | --- |
| 1 | citrate | 39 | leucine |
| 2 | citrate | 40 | leucine |
| 3 | glutamine | 41 | 2-hydroxy-3-methylvalerate |
| 4 | glutamine | 42 | unknown |
| 5 | creatinine | 43 | unknown |
| 6 | creatine | 44 | 2-hydroxybutyrate |
| 7 | lysine | 45 | 2-hydroxybutyrate |
| 8 | lysine | 46 | 2-methyl-2-oxovalerate |
| 9 | alanine | 47 | unknown |
| 10 | arginine | 48 | 1,5-anhydroglucitol |
| 11 | choline | 49 | 2,3-butanediol |
| 12 | glucose+glyceric acid+ascorbate | 50 | 3-methyl-2-hydroxybutanoic acid |
| 13 | glucose+glyceric acid+ascorbate | 51 | Alanine |
| 14 | glucose+unknown | 52 | Arabinose |
| 15 | glucose+unknown | 53 | C16:0 fatty acid |
| 16 | glucose +unknown | 54 | Citric acid |
| 17 | glucose +unknown | 55 | Glucose |
| 18 | glucose +unknown | 56 | Glutamine |
| 19 | glucose+trimethylamine N-oxide | 57 | Glycerol |
| 20 | glucose+carnitine | 58 | Lactic acid |
| 21 | glucose+carnitine | 59 | Lysine |
| 22 | glucose+phosphocholine | 60 | Mannose |
| 23 | acetone | 61 | Myo-inositol |
| 24 | acetate | 62 | Ornithine |
| 25 | lactate | 63 | Phenylalanine |
| 26 | lactate | 64 | Phosphate |
| 27 | 3-hydroxyisovaleriate | 65 | Pyruvic acid |
| 28 | 2-methyl-2-oxovalerate | 66 | Ribitol or arabitol |
| 29 | unknown | 67 | Sucrose |
| 30 | 2-oxobutyrate | 68 | Threonine |
| 31 | 2-oxobutyrate | 69 | ascorbic acid derv 1 |
| 32 | valine | 70 | butanediol isomer 2 |
| 33 | 2-oxobutyrate | 71 | erythronic acid |
| 34 | valine | 72 | fructose |
| 35 | unknown | 73 | inositol |
| 36 | valine | 74 | meso-erythritol |
| 37 | valine | 75 | sn-Glycerol-3-Phosphate |
| 38 | leucine | 76 | urea |
